# MiR-652-3p is upregulated in non-small cell lung cancer and promotes proliferation and metastasis by directly targeting Lgl1

**DOI:** 10.18632/oncotarget.7697

**Published:** 2016-02-25

**Authors:** Wenhui Yang, Chengcheng Zhou, Mei Luo, Xuejiao Shi, Yuan Li, Zengmiao Sun, Fang Zhou, Zhaoli Chen, Jie He

**Affiliations:** ^1^ Department of Thoracic Surgery, Cancer Hospital, Peking Union Medical College and Chinese Academy of Medical Sciences, Beijing 100021, China

**Keywords:** miR-652-3p, Lgl1, NSCLC, proliferation, metastasis

## Abstract

Our previous study found that miR-652-3p is markedly upregulated in the serum of patients with NSCLC and suggesting that miR-652-3p is a potential biomarker for the early diagnosis of NSCLC. In this study, we detected the expression of miR-652-3p in NSCLC tumor tissues and cell lines and investigated the effect of miR-652-3p on the proliferation and metastasis of NSCLC cells. Our results showed that the expression of miR-652-3p was significantly upregulated in tumor tissues of 50 patients with NSCLC, and it was significantly higher in patients with positive lymph node metastasis, advanced TNM stage and poor prognosis. Using functional analyses by overexpressing or suppressing miR-652-3p in NSCLC cells, we demonstrated that miR-652-3p promoted cell proliferation, migration, invasion and inhibited cell apoptosis. Moreover, the lethal(2) giant larvae 1 (Lgl1) was identified as a direct and functional target of miR-652-3p. Overexpression or knockdown of miR-652-3p led to decreased or increased expression of Lgl1 protein, and the binding site mutation of *LLGL1* 3′UTR abrogated the responsiveness of the luciferase reporters to miR-652-3p. Overexpression of Lgl1 partially attenuated the function of miR-652-3p. Collectively, these results revealed that miR-652-3p execute a tumor-promoter function in NSCLC through direct binding and regulating the expression of Lgl1.

## INTRODUCTION

Lung cancer is the most commonly diagnosed cancer and the leading cause of cancer death in men and women worldwide. An estimated 1.8 million new lung cancer cases occurred worldwide in 2012, accounting for approximately 13% of all cancer diagnoses [1]. The overall 5-year survival rate for lung cancer is only 17.1%, and it declines to 3.6% for those with stage IV disease [2]. Late diagnosis of lung cancer is an obstacle for survival. Thus, diagnosis at an early stage, understanding the molecular mechanisms and identifying novel targets are urgently needed for the treatment of NSCLC.

MicroRNAs are approximately 22 nucleotide non-coding RNAs that regulate gene expression by complementarily binding to the 3′UTR sequence of their target mRNA, thus leading to mRNA degradation or translational repression. MicroRNAs have also been implicated in transcriptional regulation by targeting promoter elements, a phenomenon known as RNA activation (RNAa) [3, 4]. MicroRNAs can function as either oncogenes or tumor suppressors involved in the initiation and progression of different types of cancer [5–7]. A number of miRNAs have been reported to have altered expression and to be involved in the carcinogenesis and development of NSCLC, thus potentially acting as biomarkers for diagnosis, prognosis, and radiotherapy and chemotherapy prediction [8–12]. Although the expression of miR-652-3p has been reported to be upregulated in human breast cancer [13], osteosarcoma [14] and rectal cancer [15], whereas downregulated in malignant pleural mesothelioma [16], the function of miR-652-3p in tumorigenesis and progression, as well as its putative target genes, are still largely unknown. In our previous study, we found that the level of miR-652-3p is significantly increased in the serum of patients with NSCLC compared with healthy individuals, suggesting that it may be a novel biomarker for NSCLC diagnosis [17].

In this study, we detected the expression of miR-652-3p in NSCLC tumor tissues and cell lines. We further investigated the role of miR-652-3p in the proliferation and metastasis of NSCLC cells. Moreover, we identified one of the target genes of miR-652-3p and demonstrated that this target gene partially mediated the function of miR-653-2p in NSCLC cells.

## RESULTS

### miR-652-3p expression is upregulated in NSCLC tumor tissues and cell lines and correlated with the disease progression of patients with NSCLC

**Figure 1:**
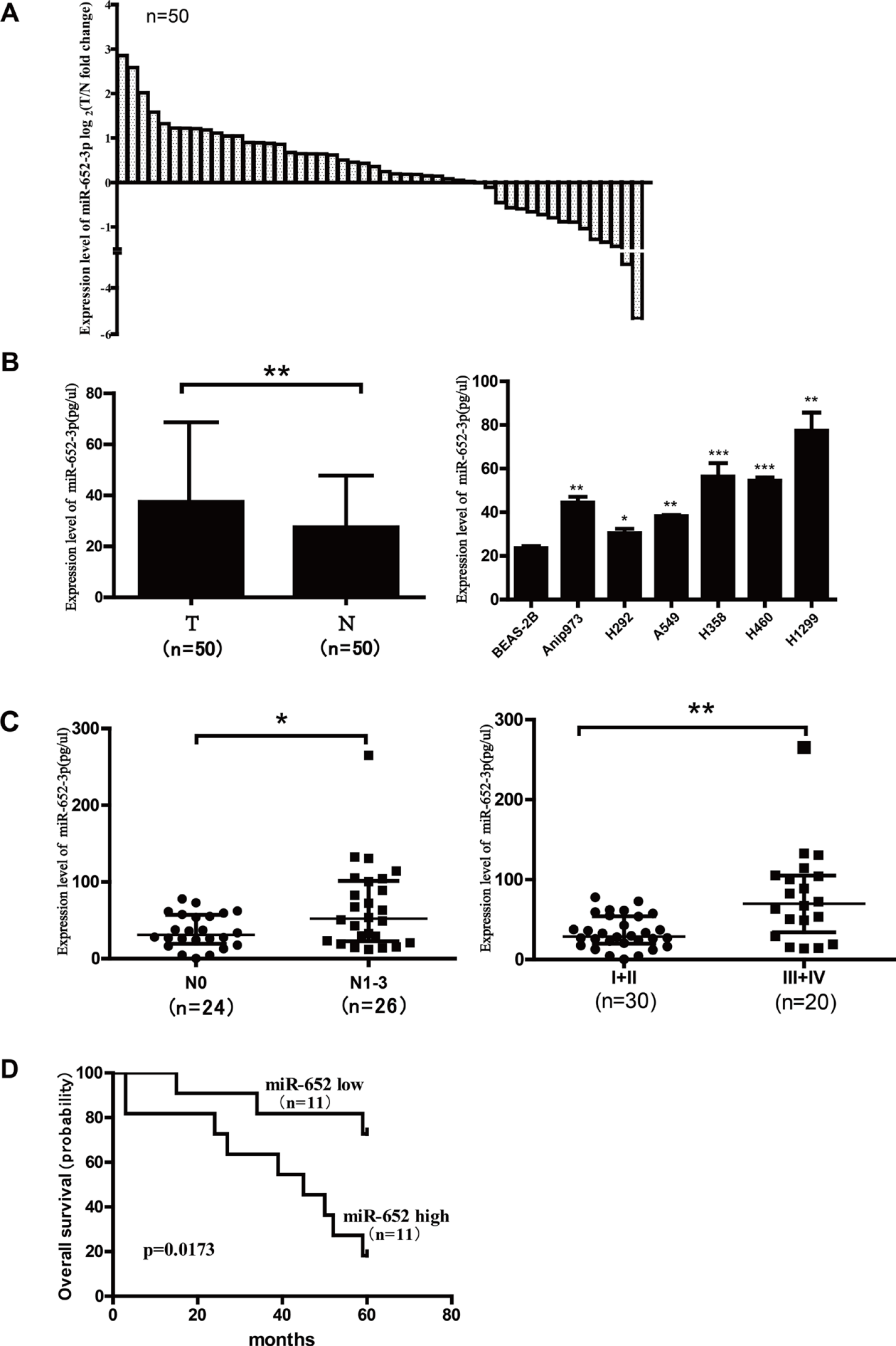
The expression of miR-652-3p is upregulated in NSCLC tissues and cell lines, and correlated with disease progression of patients with NSCLC MiR-652-3p expression was examined in paired tumor tissues (T) and matched adjacent normal tissues (N) from 50 NSCLC patients and NSCLC cell lines using qRT-PCR, the synthetic miR-652-3p mimics were reverse transcribed (RT), and then the RT product were diluted by 10-fold serial and used as template of qPCR. The standard curve was used to calculate the concentration of miR-652-3p in samples. qPCR was performed in triplicates for each sample. (**A**) The fold change of miR-652-3p expression in tumor tissues compared with adjacent normal tissues in 50 patients were shown as log2(fold change) = log2(T_miR-652-3p_/N_miR-652-3p_). Each cloumn represents a patient. (**B**) The miR-652-3p expression in 50 tumor tissues was compared with that in 50 matched normal tissues, and miR-652-3p expression in six NSCLC cell lines were compared with that in BEAS-2B cell. Median with interquartile range was shown, Mann-Whitney *U* test was used. (**C**) The expression of miR-652-3p was compared between 26 patients without lymph node metastasis (N0) and 24 patients with mediastinal lymph node metastasis (N1–N3), and between 30 patients with advanced stages (III and IV) and 20 patients with early stages (I and II). Median with interquartile range were shown, Mann-Whitney *U* test was used. (**D**) 22 NSCLC patients with their follow-up information available was classified into high expression group (*n* = 11) and low expression group (*n* = 11) by the median of miR-652-3p expression in tumor tissues. Kaplan-Meier overall survival curve of two group patients was shown, Log-rank test was used. *p < 0.05 and **p < 0.01.

First, we detected the expression of miR-652-3p in tumor tissues and adjacent normal tissues of 50 patients with NSCLC using qRT-PCR. The expression of miR-652-3p in 6 NSCLC cell lines and an immortalized human bronchial epithelial cell (BEAS-2B) were also detected. The relationship between the expression of miR-652-3p and the clinicopathologic information of patients with NSCLC was analyzed. The results showed that miR-652-3p expression was upregulated in tumor tissues compared with adjacent normal tissues in 68% (34/50) of patients with NSCLC (Figure 1A). The upregulation of miR-652-3p in tumor tissues compared with adjacent normal tissues was signifincant, and six NSCLC cell lines also showed significantly higher miR-652-3p expression than BEAS-2B cells (Figure 1B). In addition, the results showed that in patients with lymph node metastasis and advanced stages of NSCLC, the expression of miR-652-3p was significantly increased than in patients without lymph node metastasis and with early stages of NSCLC (Figure 1C). The 5-year follow-up information of 22 out of 50 patients was collected, and the survival of these 22 patients was analyzed. The Kaplan-Meier method and Log-rank test analysis found that the upregulation of miR-652-3p was significantly associated with the poor overall survival of patient with NSCLC (*p* = 0.017) (Figure 1D). These results suggested that the expression of miR-652-3p was upregulated and implicated in the progression of NSCLC.

### miR-652-3p promotes the proliferation of NSCLC cells *in vitro*

**Figure 2:**
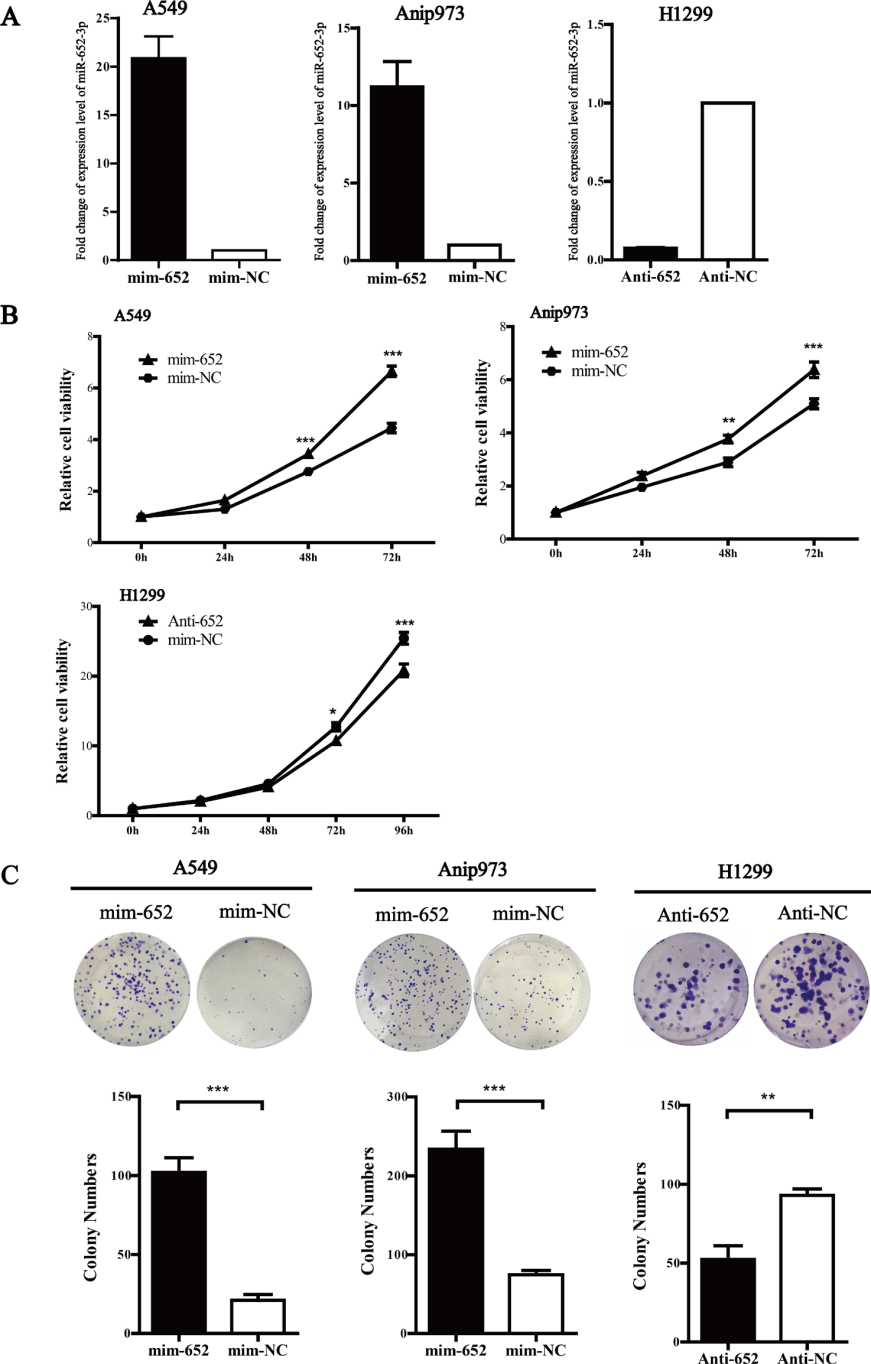
MiR-652-3p promotes cell proliferation (**A**) The miR-652-3p expression in A549 and Anip973 cells transfected with miR-652-3p mimics for 48 hours were compared with mimics negative control, and the expression of miR-652-3p in H1299 cells transfected with miR-652-3p inhibitor for 48 hours was compared with inhibitor negative control. The NC was set as 1. The column represents the mean of fold change for three independent experiments, bar represents S.E.M, each experiment performed in triplicates, *t* test was uesd. (**B**) The cell proliferation ability were compared between the miR-652-3p mimics transfection and mimics negative control in A549 and Anip973 cells, or miR-652-3p inhibitor transfection and inhibitor negative control in H1299 cells. The proliferation rate was calculated by dividing OD_450_ of 24, 48 and 72 hours by that of 0 hours after transfection. The difference of proliferation rate in 24, 48 and 72 hours between groups were copmared, point represents mean of three independent experiments, bar represents S.E.M, each experiment performed in triplicates, *t* test was uesd. (**D**) The colony formation ability was compared between miR-652-3p mimics or inhibitor transfection and negative control group, and the number of clones were counted. Column, mean of three independent experiments, bar, S.E.M, each experiment performed in triplicates, *t* test was uesd. *p < 0.05, **p < 0.01 and ***p < 0.001.

To investigate the biological function of miR-652-3p in NSCLC cells, we selected A549 and Anip973 cells with relatively lower endogenous miR-652-3p for transfection with miR-652-3p mimics, whereas H1299 with the relatively higher endogenous miR-652-3p for miR-652-3p inhibitor transfection. After transfection, the miR-652-3p mimics significantly increased the level of mature miR-652-3p in A549 and Anip973 cells, and anti-miR-652-3p markedly decreased the level of mature miR-652-3p in H1299 cells (Figure 2A). Overexpression of miR-652-3p significantly improved the proliferation of A549 and Anip973 cells 48 hours after transfection, whereas knockdown of miR-652-3p significantly inhibited the proliferation of H1299 cells 72 hours after transfection (Figure 2B). The colony formation assay showed similar results in which altered expression of miR-652-3p significantly impacted the proliferation ability of NSCLC cells (Figure 2C).

### miR-652-3p suppresses the apoptosis of NSCLC cells

**Figure 3:**
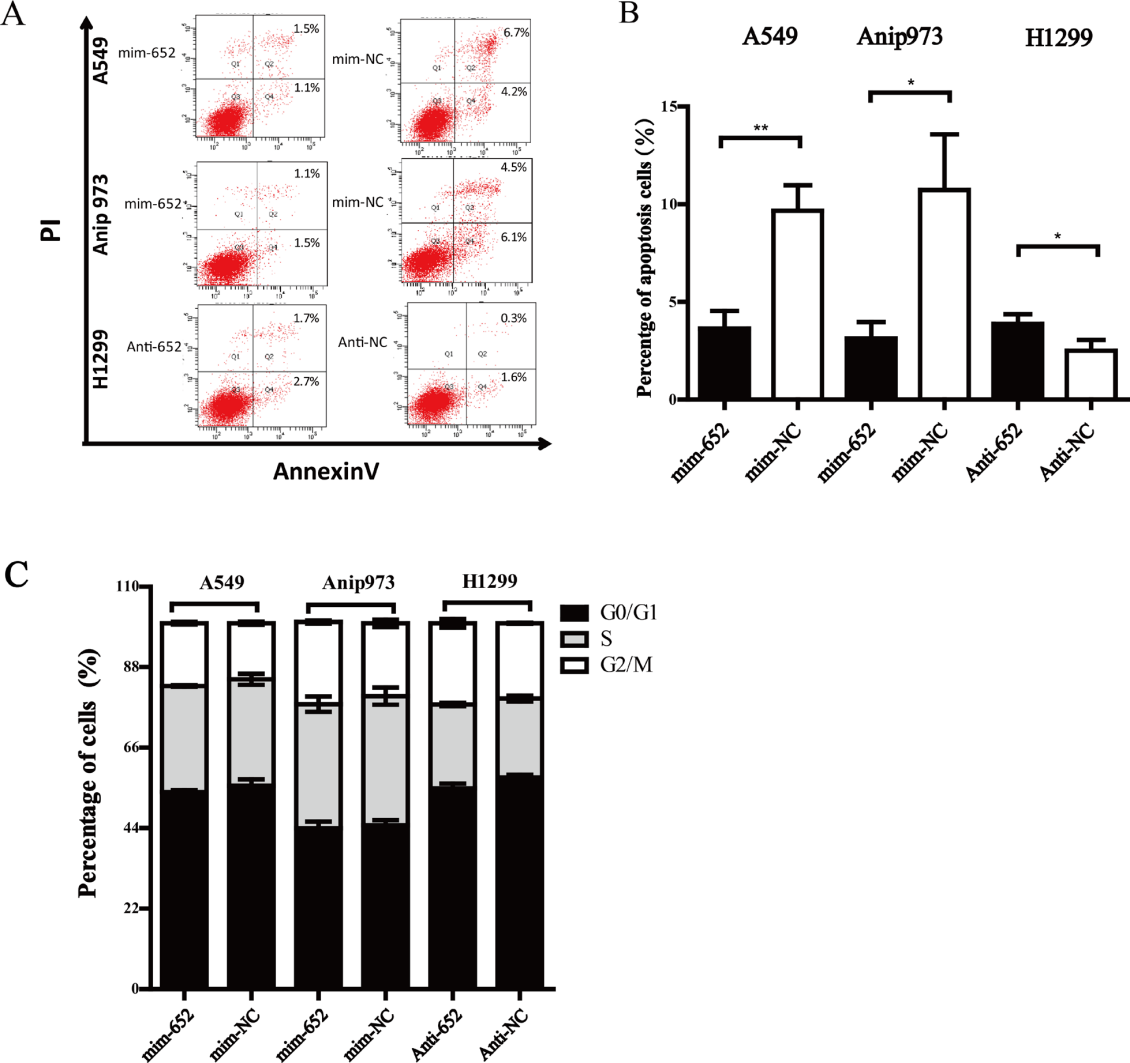
MiR-652-3p inhibits cell apoptosis (**A**) The percentage of early and late apoptosis cells was shown. (**B**) The difference of percentage of early plus late apoptosis cells were compared between miR-652-3p mimics or inhibitor transfection and negative control group. (**C**) The percentage of cell cycle between miR-652-3p mimics or inhibitor transfection and negative control group were shown., no significant difference was found. Column, mean of three independent experiments, bar, S.E.M, each experiment performed in triplicates, *t* test was uesd. *p < 0.05 and **p < 0.01.

To further investigate the mechanism of how miR-652-3p regulating the proliferation of NSCLC cell, we analyzed the effect of miR-652-3p on cell cycle and apoptosis using a flow cytometry assay. The results showed that mim-miR-652-3p significantly inhibited the apoptosis of A549 and Anip973 cells, while anti-miR-652-3p markedly increased the apoptosis of H1299 cells (Figure 3A, 3B). However, no significant difference of cell cycle were observed after the upregulation or knockdown of miR-652-3p in NSCLC cells (Figure 3C). These results suggested that miR-652-3p regulated cell proliferation mainly through suppressing the apoptosis of NSCLC cells.

### miR-652-3p promotes migration and invasion of NSCLC cells

**Figure 4:**
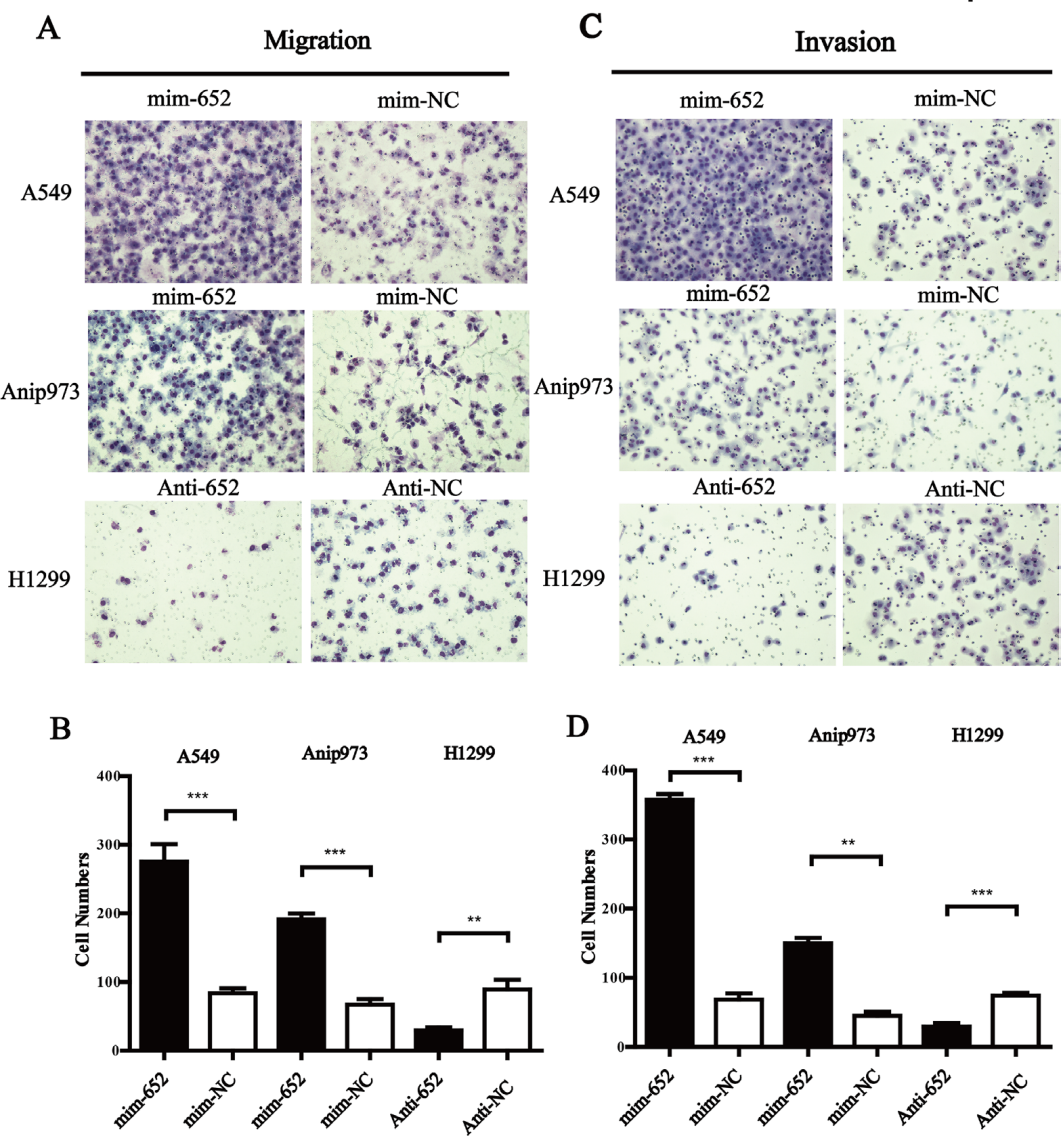
MiR-652-3p promotes the migration and invasion of NSCLC cells Twenty-four hours after miRNA transfection, serum was removed and cells were cultured in serum-free medium for 12 hours. Cells were plated into the upper chamber of 24-well transwell inserts uncoated or coated with Matrigel for migration or invasion assay for 12–24 hours. The cells on the lower side of the chamber without Matrigel (**A**) or coated with Matrigel (**C**) were fixed, stained and counted in three different areas at 100-fold magnification. The cell numbers of migration (**B**) and invasion (**D**) were compared between goups. Column, mean of three independent experiments, bar, S.E.M, each experiment performed in triplicates, *t* test was uesd. **p < 0.01 and ***p < 0.001.

Given the positive relationship of upregulated expression of miR-652-3p and lymph nodes metastasis, we further evaluated the effect of miR-652-3p on the migration and invasion ability of NSCLC cells. The results showed that mim-miR-652-3p significantly increased the migration and invasion ability of A549 and Anip973 cells (Figure 4A, 4B), while anti-miR-652-3p significantly suppressed the migration and invasion ability of H1299 cells (Figure 4C, 4D).

### Lgl1 is a direct target of miR-652-3p in NSCLC cells

**Figure 5:**
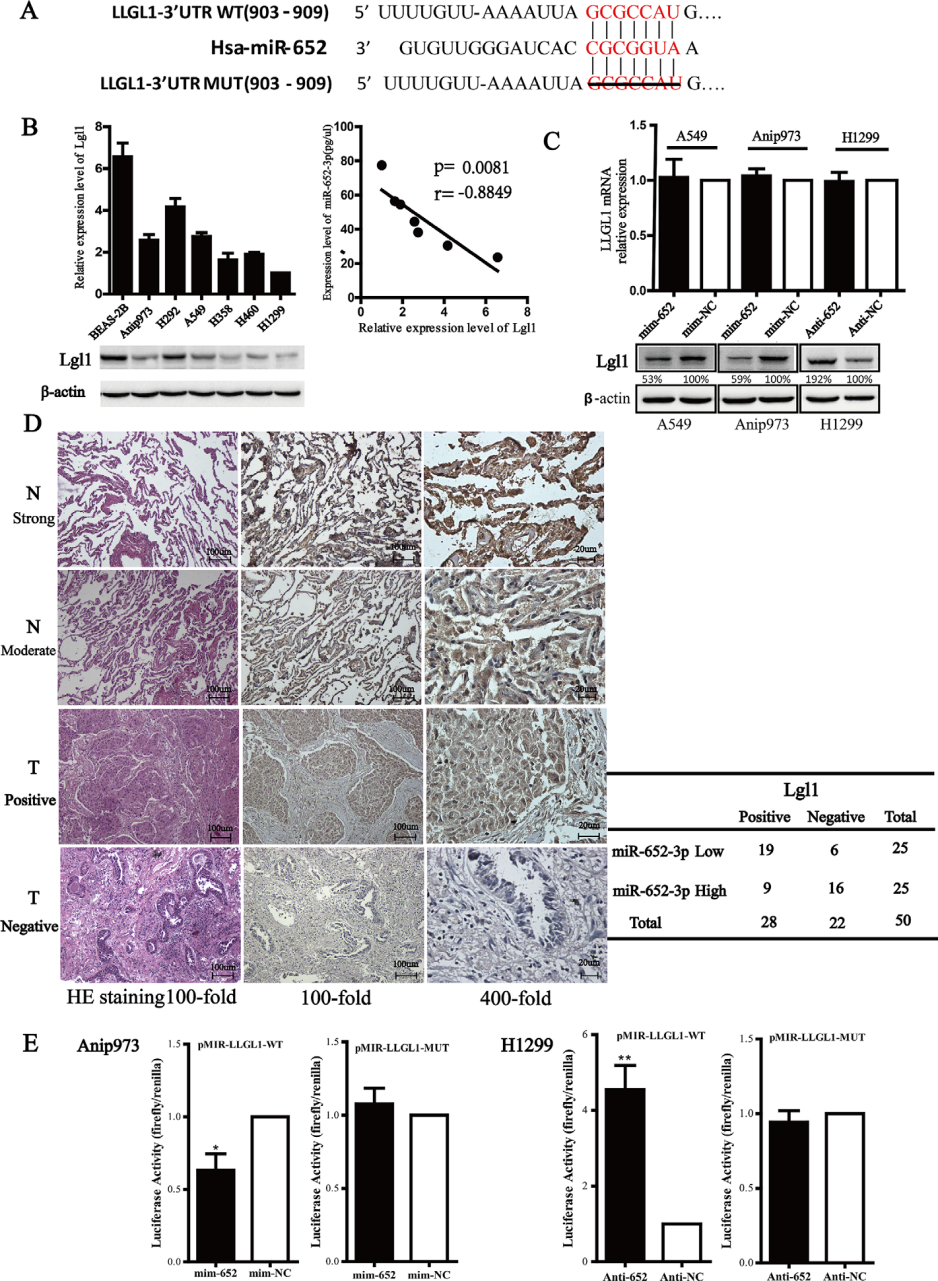
Lgl1 is a direct target of miR-652-3p (**A**) The three public miRNA databases (miRDB, miRanda and DIANA) predicted that *LLGL1* might be a target for miR-652-3p, and the 3′-UTR of *LLGL1* mRNA contains a highly conserved and perfect complementary region from position 903 to 909 for biding the seed sequence of miR-652-3p. A 112 nt nucleotide sequence of *LLGL1* mRNA 3′UTR containing the predictive miR-652-3p binding seed sequence were synthesized and inserted to construct pMIR-*LLGL1*-WT, and the truncated mutation of the above nucleotide sequence lacking the 7 nt seed sequence were inserted to construct the pMIR-*LLGL1*-Mut vector. (**B**) The total proteins were isolated from 6 NSCLC cells and a normal bronchus epithelial cell line for Lgl1 detection by Western blotting, β-actin was used as the loading control. The gray density was quantified, and the relative expression was calculated by dividing the Lgl1 by β-actin. The correlation of Lgl1 expression and miR-652-3p level was ananlyzed using Pearson's chi-square test. Column, mean of three independent experiments, bar, S.E.M. (**C**) The level of *LLGL1* mRNA was examined using qRT-PCR in A549 and Anip973 cells transfected with miR-652-3p mimics or mim-NC as well as in H1299 cells transfected with the miR-652-3p inhibitor or anti-NC for 48 hours. GAPDH mRNA was used as an internal control. The NC was set as 1. Column, mean of three independent experiments, bar, S.E.M, each experiment performed in triplicates, *t* test was uesd (top). The Lgl1 protein level was detected by Western blotting, the gray density and relative expression level was shown (bottom). (**D**) The expression of Lgl1 in 50 tumor tissues (T) and adjacent normal tissues (N) was examined by IHC (left). The correlation between miR-652-3p level and Lgl1 expression in tumor tissues was analyzed by Fisher's exact test (right). (**E**) Cells were transfected with miR-652-3p mimics in A549 cells or inhibitor in H1299 cells for 24 hours followed by transfection with pMIR-*LLGL1*-Wt/Mut reporter plasmids along with phRL-TK. Luciferase activity was measured using a dual-luciferase reporter assay. The relative luciferase activity was calculated by LUC_firefly_/LUC_renilla_, the fold change of mimics or inhibitor compare to negative control was shown. The NC was set as 1. Column, mean of three independent experiments, bar, S.E.M, each experiment performed in triplicates, *t* test was uesd. *p < 0.05 and **p < 0.01.

We next identified potential target genes of miR-652-3p using bioinformatics algorithms, miRDB, miRanda and DIANA. All three of them predicted that *LLGL1* was one of the predicted target genes of miR-652-3p. Lgl1 protein (encoded by *LLGL1* gene) has been reported to exert tumor suppressor effects in esophageal cancer and colorectal cancer [18, 19]. The 3′UTR of the *LLGL1* mRNA contains a highly conserved and perfect complementary region from position 903 to 909 for binding the seed sequence of miR-652-3p (Figure 5A). To determine whether *LLGL1* was the target of miR-652-3p, the relationship between them was analyzed. The result showed that there was a significantly inverse relationship between miR-652-3p expression and Lgl1protein level in the 6 NSCLC cell lines and BEAS-2B cells (*p* = 0.008) (Figure 5B). Then we measured the levels of *LLGL1* mRNA and protein levels after altering the expression of miR-652-3p. The results showed that the level of Lgl1 protein was markedly decreased 72 hours after transfection of mim-miR-652-3p in A549 and Anip973 cells, whereas anti-miR-652-3p led to a significant increase of Lgl1 protein in H1299 cells. However, the level of *LLGL1* mRNA was not altered after transfection of mim-miR-652 or anti-miR-625 in NSCLC cells (Figure 5C). To further judge the relationship between Lgl1 prtotein and miR-652-3p in NSCLC tumor tissues, the level of Lgl1 protein was detected by IHC in the 50 tumor tissues and adjacent normal tissues of NSCLC. The result showed that the level of Lgl1 was markedly decreased in tumor tissues compared to that in adjacent normal tissues. In 50 tumor tissues, 22 were scoed as Lgl1 negative expression, 28 were scored as weak to moderate Lgl1 expression. However, in 50 paired normal tissues, 35 were scored as strong Lgl1 expression and 15 as moderate expression. Moreover, in tumor tissues with higher miR-652-3p levels showed lower Lgl1 protein expression and vice versa (*p* < 0.01) (Figure 5D). These results indicated that *LLGL1* is a potential target gene of miR-652-3p and the expression of Lgl1 was regulated by miR-652-3p.

We next constructed the pMIR-*LLGL1*-Wt and pMIR-*LLGL1*-Mut luciferase reporter plasmids, which contain the wild-type *LLGL1* 3′UTR sequence and the 3′UTR with the 7 nt binding site sequence deletion, respectively. Overexpression of miR-652-3p significantly decreased the luciferase activity of Anip973 cells transfected with pMIR-*LLGL1*-Wt compared to the miRNA mimic negative control, while the deletion mutation of the binding sites eliminated the effect of miR-652-3p on luciferase activity. In addition, knockdown of miR-652-3p markedly increased the luciferase activity in H1299 cells transfected with pMIR-*LLGL1*-Wt, and the mutation abrogated the effect of miR-652-3p inhibitor on the luciferase activity (Figure 5E). These results revealed that miR-652-3p regulates the expression of Lgl1 by directly binding to the *LLCL1* 3′UTR sequence in NSCLC cells.

### Lgl1 reverses the effect of miR-652-3p on the migration and invasion of NSCLC cells

**Figure 6:**
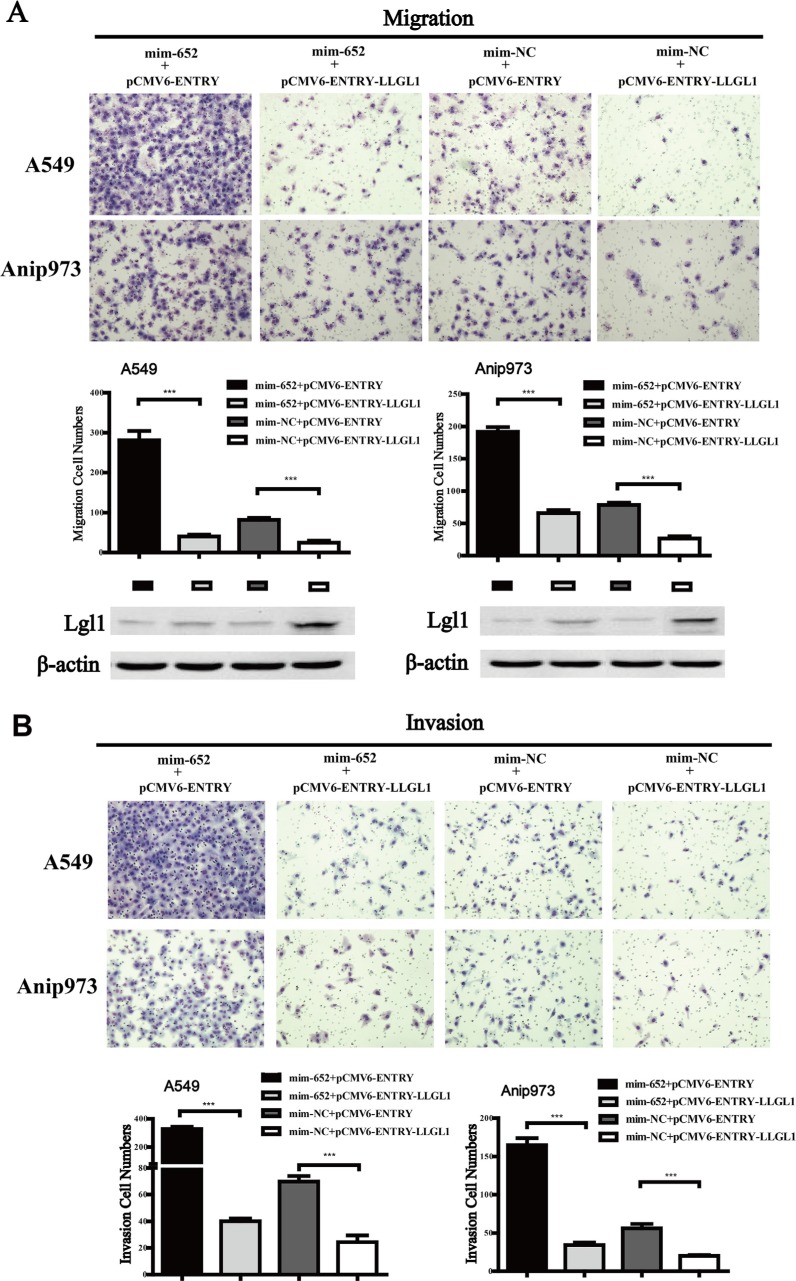
Reexpression of Lgl1 reverses the effect of migration and invasion induced by miR-652-3p (**A**) The miR-652-3p-induced migration of NSCLC cells was restored via Lgl1 reexpression. (**B**) The miR-652-3p-induced invasion of NSCLC cells was restored via Lgl1 reexpression. The cell numbers of migration and invasion were compared between goups. Column, mean of three independent experiments, bar, S.E.M, each experiment performed in triplicates, *t* test was uesd. The expression of Lgl1 protein was detected by Western blotting, β-actin was used as the loading control. ***p < 0.001.

To further investigate whether the biological function of miR-652-3p was mediated by Lgl1, we restored the expression of Lgl1 by transfecting the pCMV6-ENTRY-*LLGL1* plasmid containing the *LLGL1* open reading frame (ORF) lacking the 3′UTR sequence into A549 and Anip973 cells 24 hours after transfection with mim-miR-652-3p. The results showed that overexpression of Lgl1 significantly inhibited the migration and invasion ability of A549 and Anip973 cells compared to the pCMV6-ENTRY negative control. In addition, reexpression of Lgl1 significantly abrogated the activity of miR-652-3p in promoting the migration and invasion of A549 and Anip973 cells (Figure 6). These results suggested that the upregulated expression of miR-652-3p in NSCLC tumor tissues promotes the metastasis of NSCLC cells partially by binding and suppressing the expression Lgl1, a target gene of miR-652-3p.

## DISCUSSION

Recent reports have suggested that miRNAs not only negatively regulate gene expression at the post-transcriptional level but also positively regulate gene expression by targeting promoter elements [3, 20]. Dysregulation of miRNAs has been reported to be closely associated with oncogenesis and tumor progression in diverse cancers including lung cancer. Recently, several miRNAs have been reported to be associated with lung cancer. MiR-95-3p, miR-125a-3p, miR-30d-3p, miR-526b, miR-195 and miR-1238 have been shown to suppress the development of NSCLC [21–26], whereas miR-183 and miR-25 promote the development of NSCLC [27, 28]. However, the effects of miR-652-3p on tumor initiation and progression are still largely unknown. MiR-652-3p has recently been identified as a tumor-related gene. MiR-652-3p is upregulated in breast cancer, osteosarcoma and rectal cancer, but it is downregulated in malignant pleural mesothelioma [13–16]. However, all of these reports were based on miRNA microarray or qRT-PCR results, and identified it as a potential diagnostic biomarker without investigating the function of miR-652-3p in tumorigenesis. Our previous study also found that the level of miR-652-3p was significantly upregulated in the serum of patients with NSCLC [17]. In the present study, we further demonstrated that the expression of miR-652-3p was markedly upregulated in NSCLC tumor tissues and cell lines, and its expression was associated with the disease progerssion and poor survival of NSCLC patients. However, only 22 patients with the follow-up information were available in our study, the prognostic value of miR-652-3p in NSCLC need to be further validated in the larger samples. Furthermore, the function study showed that miR-652-3p promoted cell growth, migration and invasion and inhibited cell apoptosis. These results suggested that miR-652-3p acted as an oncogene in NSLCL and the upregulation of miR-652-3p contributes to the progression and metastasis of NSCLC. Targeting miR-652-3p as a potential treatment for NSCLC need to be further studied by *in vivo* assay.

Lethal (2) giant larvae (Lgl) proteins was first identified in Drosophila, plays a crucial role in regulating cell polarity, which is crucial for a multitude of cellular fates, including differentiation, proliferation, migration, adhesion and transformation [29, 30]. In human, the homologues of Lgl protein include Lgl1 and Lgl2 (encoded by *LLGL1* and *LLGL2* gene, respectively), which have the similar WD40 domains acting as a scaffold for coordination of multiprotein complex assemblies and involved in a wide variety of cell biological processes, including signal transduction, vesicle trafficking, cytoskeleton assembly and cell division [31, 32]. Metastasis is the major cause of death in cancer patients, and the loss of apical-basal polarity in epithelial cells and an epithelial-to-mesenchymal transition are two hallmarks of aggressive and invasive cancers [38]. Accumulating evidence has shown that Lgl1 plays a suppressive role in various human epithelial cancers. Lgl1 promotes the cell adhesion and inhibites cell migration by downregulating the expression of MMP2 and MMP14, and re-expressing of E-cadherin in melanoma cells [34]. Lgl1 induces growth suppression and apoptosis in esophageal carcinoma cells by activating the mitochondria-related pathway [18]. The expression of Lgl1 is reduced in a number of cancers, including breast cancers, lung cancers, prostate cancers, ovarian cancers, colorectal cancers, melanomas, endometrial cancers and hepatocellular carcinomas, and a significant correlation has been found between the loss of Lgl1 protein and disease progression and lymph node metastasis [19, 34, 35, 39, 40]. It was reported that *LLGL1* mRNA is frequently mutated by aberrant splicing, indicating that *LLGL1* mutation may be involved in progression of hepatocellular cancer [41]. In glioblastoma, Lgl1 is inactivated when it is phosphorylated by PKCι, which occurs as a downstream consequence of *PTEN* loss [33, 42]. However, the mechanism of Lgl1 dysregulation in lung cancer is unknown. Our study reveals a novel mechanism by which Lgl1 expression is regulated. We found that *LLGL1* mRNA was directly targeted by miR-652-3p and the Lgl1 protein level was reduced by miR-652-3p in NSCLC cells. Furthermore, reexpreesion of Lgl1 attenuated invasion and migration promoting effect induced by miR-652-3p, indicating that Lgl1 functions as a mediator of the effect of miR-652-3p on metastasis. Whether the dysregulation of Lgl1 is regulated by other mechanisms than miRNA targeting in NSCLC is not yet known. Imamura *et al.* reported that Lgl2, the homologous protein of Lgl1 in human, was co-locaolized and interacted with the aPKC lambda/iota in cell apical membrane of lung adenocarcinoma tissues by double-immunohistochemical analysis, which raised a possibility of phosphorylation of Lgl2 by aPKC λ/ι in lung cancer [43]. However, whether the activity of Lgl1 is regulated by phosphorlation associated with PKC in NSCLC need to be further studied.

In conclusion, we provided clear evidence that miR-652-3p is frequently upregulated in NSCLC tissues, and miR-652-3p execute a tumor-promoter function in NSCLC. We also revealed a new regulatory mechanism of Lgl1 in NSCLC, as it is downregulated by miR-652-3p through direct targeting of its 3′UTR.

## MATERIALS AND METHODS

### Patients and tissue samples

We retrospectively enrolled 50 patients who underwent the curative resection for NSCLC at the Cancer Hospital of Chinese Academy of Medical Sciences between January 2010 and May 2013, none of them received preoperative radiotherapy or chemotherapy. The frozen stored specimens of these 50 pairs of tumor tissues and adjacent non-tumor tissues were obtained for miRNA extraction and qRT-PCR assay, the formalin fixed, and paraffin-embedded (FFPE) specimens of these 50 pairs of tissues were obtained for immunohistochemistry (IHC) assay. The clinicopathological information of these 50 patients were reviewed, and pTNM stage of NSCLC was classified according to the seventh edition of the American Joint Committee on Cancer staging system. The follow-up information of 22 among 50 patients with NSCLC were obtained for the survival analysis. Written informed consents were signed by all the patients enrolled in this study. The study was approved by the Institutional Review Board of Cancer Hospital of Chinese Academy of Medical Sciences and conducted according to the guidelines approved by the ethics committee.

### Cell culture and transfection

Six human NSCLC cell lines (A549, H1299, H460, H358, H292 and Anip973) were cultured in RPMI1640 medium (Hyclone), and immortalized human bronchial epithelial cell (BEAS-2B) were cultured in DMEM medium (Hyclone), supplemented with 10% FBS (Gibco) and 0.2% penicillin-streptomycin (Hyclone). The miR-652-3p mimics (mim-miR-652), miR-652-3p inhibitor (anti-miR-652), negative control miR mimics and miR inhibitor (mim-miR-NC and anti-miR-NC) were purchased from Ambion and transfected at a final concentration of 30 nM with Lipofectmine 2000 (Invitrogen).

### RNA extraction and qRT-PCR

Total RNA was extracted from frozen tissues (30 mg tissue of each sample) and cultured cells using a miRNeasy Minikit (QIAGEN), and the quality and concentrations of total RNA were measured by Nanodrop 2000c. 1 μg total RNA were used for the first strand cDNA synthesis in a 15 μl reaction system using a TaqMan MicroRNA Reverse Transcription kit (Thermo Fisher), and 1 μl synthesized cDNA was then amplified in 10 μl reaction system using TaqMan gene expression master mix (Thermo) and Applied Biosystems 7900 Real Time PCR system following the manufacturer's instructions. The miR-652-3p specific stem-loop primers were purchsed from Thermo Fisher (Cat. 4427975). The synthetic miR-652-3p mimics with the identical sequences as the mature miR-652-3p were purchsed from Invitrogen. 150 ng miR-652-3p mimics was used for reverse transcription in 15 μl reaction system. The concentration of reverse transcription product was measured and the serial diluted reaction product (10 ng/μl, 1 ng/μl, 0.1 ng/μl, 10 pg/μl and 1 pg/μl) were used as the template of qPCR. The standard curve was used to calculate the concentration of miR-652-3p in samples.

The levels of *LLGL1* mRNA transcript were measured by RevertAid^™^ First strand cDNA Synthesis kit and Power SYBR^®^ Green PCR Master Mix (Thermo) using a forward primer (5′-GCTGCTTCGATCCCT ACAGTGAC-3′) and reverse primer (5′-CGGCACATCCT AAGCTCCAG-3′). GAPDH was used as an internal control, and GAPDH was amplified with a forward primer (5′-CCTGGTATGACAACGAATTTG-3′) and a reverse primer (5′-CAGTGAGGGTCTCTCTCTTCC-3′). All the qPCR reaction was performed in triplicate.

### Cell proliferation and colony formation assays

For the proliferation assay, 1 × 10^3^ cells/well were plated in 96-well plates, 24 hours later, mim-miR-652-3p or anti-miR-652-3p were transfected, and then cell viability were tested every 24 hours for 96 hours using the cell counting kit-8 (CCK-8; Dojindo). To assess the colony formation ability, transfected cells (400 cells/well) were seeded into 6-well plates and maintained in media containing 10% FBS. After 2 weeks, the colonies were fixed in methanol, stained with 0.1% crystal violet (Sigma) and the clone number were counted using Quantity One software.

### Cell cycle analysis

The cell cycle analysis was performed using a cell cycle detection kit (KGA). Sixty hours after mim-miR-652-3p or anti-miR-652-3p transfection, cells were cultured in medium removal of FBS for 12 hours to synchronize. Then cells were fixed in 70% cold ethanol at 4°C for 24 hours and then stained with 50 μg/ml propidium iodide (PI) for 30 min. The cell cycle distribution was analyzed by Coulter Epics XL flow cytometry (Becton Dickinson FACSCanto II). The distribution of cells in distinct cell cycle phases was determined using ModFit LT3.2 (Verity Software House).

### Cell apoptosis assay

Apoptosis was measured using the BD Pharmingen FITC Annexin V apoptosis detection kit I (BD Biosciences). Forty-eight hours after mim-miR-652-3p or anti-miR-652-3p transfection, 5 × 10^5^ Cells were incubated with Annexin V-fluorescein isothiocyanate (FITC) and PI, and the percentage of apoptotic cells was analyzed by flow cytometry (Becton Dickinson).

### Migration and invasion assays

Twenty-four hours after miRNA transfection, serum was removed and cells were cultured in serum-free medium for 12–24 hours. Cells (5 × 10^4^ or 1 × 10^5^) in serum-free RPMI 1640 medium were plated into the upper chamber of 24-well transwell inserts (Corning, 8.0 μm pores) that were either uncoated or coated with Matrigel (BD Biosciences) for migration or invasion assay, the cells were then allowed to translocate toward medium containing 20% FBS for 12–24 hours. The cells on the lower side of the chamber were fixed, stained and counted in three different areas at 100-fold magnification. pCMV6-ENTRY-*LLGL1*, which contains the *LLGL1* open reading frame (ORF) lacking the 3′UTR sequence, was purchased (OriGene Technologies) and was used to restore the miR-652-3p-resistant Lgl1 expression. Twenty-four hours after mim-miR-652 transfection, cells were transfected with pCMV6-ENTRY-*LLGL1* or the control vector pCMV6-ENTRY (OriGene). Twenty-four hours later, cells were digested and seeded for the migration and invasion assay as described above.

### Western blot and immunohistochemistry

Seventy-two hours after transfection, cells were lysed with RIPA lysis buffer (Santa Cruz). Western blot analysis then was performed [44]. An anti-Lgl1 antibody (1:1000, Cell Signaling Technology), an anti-β-actin antibody (1:10000, Santa Cruz), a HRP-labeled goat anti-rabbit IgG and an anti-mouse IgG (Abgent) were used. IHC was performed on 4 μm FFPE sections and stained with hematoxylin and eosin or treated with anti-Lgl1 antibody (1:400, ATLAS, HPA023569). The IHC staining score were calculated by combining the percentage of positive cells and staining intensity by two pathologists independently. The staining intensity was scored as: 0 (negative), 1 (weak), 2 (moderate), and 3(strong). The percentage of positive cells was assigned as the following categories: 0 (< 5%), 1 (5–25%), 2 (26–50%), 3 (51–75%), or 4 (> 75%). The two scores were then multiplied to produce a weighted score for each sample: negative (0), weak (1–4), moderate (5–8), strong(9–12). For the subsequent analysis, weak, moderate and strong starining were classified as positive group.

### Luciferase reporter assay

The pMIR plasmid (Ambion) was used to construct the reporter plasmid. A 112 nt nucleotide sequence of *LLGL1* mRNA 3′UTR containing the binding site for miR-652-3p seed sequence were synthesized and inserted into the Mlu1 and SacI site to construct pMIR-*LLGL1*-WT, and the truncated mutation of the above nucleotide sequence lacking the 7 nt seed sequence were inserted to construct the pMIR-*LLGL1*-Mut vector. The Renilla luciferase expression construct phRL-TK was used as an internal control (Promega). After transfection with miRNA mimics or inhibitors for 24 hours, pMIR-*LLGL1*-Wt/-Mut (200 ng per well in 24-well plate) along with control phRL-TK (2 ng per well) were transfected into the cells. After 24 hours, the cells were harvested and lysed with passive lysis buffer. Luciferase activity was measured by a dual-luciferase reporter system (Promega) using LB 960 Centro (Berthold). The luminescence intensity of Firefly luciferase was normalized to that of Renilla luciferase.

### Statistical analysis

Statistical analyses were performed using SPSS 19.0 software. The difference of miRNA expression between groups was evaluated by the Mann-Whitney *U* test. The Kaplan-Meier method Log-rank test was used for survival analysis. The results of cell experiments were presented as means and S.E.M from three independent experiments, and the differences among groups were analyzed by unpaired Student's *t* test. Pearson correlation analysis was used to analyze the association between the expression of miR-652-3p and Lgl1 in NSCLC cells. The Fisher's exact test was used to analyze the association between the expression of miR-652-3p and Lgl1 in tumor tissues. Differences were considered significant at *p* < 0.05.
